# Comparison of the prevalence rates of HIV infection between men who have sex with men (MSM) and men in the general population in sub-Saharan Africa: a systematic review and meta-analysis

**DOI:** 10.1186/s12889-019-8000-x

**Published:** 2019-12-04

**Authors:** P. H. Septime Hessou, Yolaine Glele-Ahanhanzo, Rheda Adekpedjou, Carin Ahouada, R. Christian Johnson, Michel Boko, Hervé Tchala Vignon Zomahoun, Michel Alary

**Affiliations:** 1 Centre National de Référence de Recherche et de Prise en Charge du Sida (CNRRPEC-CNHU/Bénin), Cotonou, Benin; 2 Institut Régional de Santé Publique (IRSP), Université d’Abomey-Calavi (UAC), Ouidah, Bénin; 3 Centre de Recherche sur les Soins et Services de Première Ligne de l’Université Laval (CERSSPL-UL), Université Laval, Québec, Canada; 4 Axe Santé des populations et pratiques optimales en santé Centre de Recherche du CHU de Québec, Université Laval, Hôpital du Saint-Sacrement, Quebec City, Canada; 5 Centre Inter-facultaire de Formation et de Recherche en Environnement pour le Développement (CIFRED), Université d’Abomey-Calavi (UAC), Calavi, Benin

**Keywords:** HIV, Men who have sex with men (MSM), Prevalence, Sub-Saharan Africa

## Abstract

**Background:**

According to the 2015 report of the Joint United Nations Program on Human Immunodeficiency Virus (HIV) and Acquired Immune Deficiency Syndrome (AIDS), the prevalence rates of HIV infection among men who have sex with men (MSM) varied from 6 to 37% depending on the country, far exceeding the national prevalence rates. The present study on HIV infection among men who have sex with men in sub-Saharan Africa was conducted to describe the different sampling methods used to identify this target population and compare the prevalence rates of HIV infection among MSM to that of men in the general population.

**Methods:**

The selection of studies to be included was carried out in the principal electronic databases. The 2009 Preferred Reporting Items for Systematic Reviews and Meta-Analyses (PRISMA) directives were used throughout the entire process. Bias evaluation was performed using the Mixed Methods Appraisal Tool. For each country, HIV prevalence values in both groups were calculated. A prevalence ratio was also calculated to compare the prevalence rates of the two groups.

**Results:**

Seventeen articles were selected. Most of the studies (82.35%) used the Respondent-Driven Sampling method. The average prevalence rate was 17.81% (range: 3.7–33.46) for MSM and 6.15% (range: 0.5–19.7) for men in the general population. Overall, the human HIV prevalence rate was 4.94 times higher among MSM than among men in the general population (95%CI: 2.91–8.37). The western and central regions of Africa, as well as low-prevalence countries (prevalence < 1%), had very high prevalence ratios: 14.47 (95% CI: 9.90–21.13) and 28.49 (95% CI: 11.47–72.71), respectively.

**Conclusion:**

MSM are at higher risk of HIV infection than men in the general population. The prevalence ratios are particularly elevated in West and Central Africa as well as in low-prevalence countries. Close monitoring of the situation, research and preventive measures are essential to control the epidemic amongst MSM.

## Background

Unprotected sex between men facilitates the transmission of sexually transmitted infections (STIs) and human immunodeficiency virus (HIV) [1]. Unprotected anal penetration is high-risk behaviour for HIV transmission. In the 2015 UNAIDS (Joint United Nations Program on HIV/AIDS) report, studies in sub-Saharan Africa found prevalence rates of HIV infection ranging from 6 to 37% among MSM. As the observed prevalence rate of HIV in the general population was between 0.1 and 19%, the prevalence rate among men who have sex with men (MSM) was often 13 to 17 times higher [2].

In 2015, according to the progress reports on the global AIDS (Acquired Immune Deficiency Syndrome) response, the highest prevalence rates of HIV infection among MSM were as follows: 19% in central and western Africa; 15% in southern and eastern Africa; 12% in Latin America; 11% in the Asia-Pacific region; and 8% in central and western Europe and North America [2–4]. Although data existed on the prevalence of HIV infection among MSM in countries in sub-Saharan Africa, very few systematic reviews have been conducted to allow for a comprehensive summary of the prevalence data, to measure the extent of this disease among MSM and to compare it to that of the general population [3, 5]. The only systematic review of this type that we identified was published 12 years ago and was not specific to sub-Saharan Africa [6]. Moreover, although much more attention has been given to African MSM since the publication of this review, access to MSM in many countries in sub-Saharan Africa remains generally difficult, particularly in terms of their potential participation in epidemiological studies. This is due to discrimination and/or criminalization of their sexual orientation, as well as the social stigma associated with their behaviour. Few or no literature reviews have identified the different methods used to sample this population in epidemiological studies in sub-Saharan Africa [1]. A comparison in sub-Saharan Africa will help MSM emerge from the shadows. It will highlight their heavy burden in terms of new HIV infections and draw the attention of public authorities to the need to take this target into account in intervention programs in a hostile environment. Thus, the purpose of our systematic review is twofold: to compare the prevalence of HIV infection among MSM and men in the general population, and to describe the various sampling methods used to reach this “hidden” MSM population in sub-Saharan Africa.

## Methods

This systematic review was conducted in accordance with the Preferred Reporting Items for Systematic Reviews and Meta-Analyses (PRISMA) statement [7].

### Inclusion criteria

The inclusion criteria for the studies were as follows: (1) the study population comprised MSM populations in sub-Saharan Africa aged 18 years and over; (2) exposure was defined as having voluntary and consensual sexual intercourse with a man at least once in the 12 months prior to the study; (3) the outcome sought was an HIV infection rate whose frequency measurement was expressed as prevalence and was based on actual HIV tests conducted in the course of the study; (4) they took place in a country where Demographic Health Surveys (DHS) provided information on the prevalence of HIV infection for men from the general population within a few years of the MSM study; and (5) the study design was cross-sectional or had a cross-sectional component of recruitment in a longitudinal study. General population HIV prevalence estimates for a given country were all based on contemporary DHS surveys in the same country. The latter included all men, and therefore could include some MSM. There were no period restrictions or year limitations. All studies with a sample size of less than 50 subjects and/or self-reported HIV infection were excluded. We found that studies with sample sizes of less than 50 subjects were not sufficiently precise to be included and we excluded those with self-reported HIV status to avoid information bias, be more specific and facilitate comparisons.

### Data source and search strategy

The search was carried out using the following electronic databases: PubMed, EMBASE, Cochrane, Web of Science, Scopus and Google Scholar. The PICOS (Population Intervention Comparison Outcomes Study design) approach was used in this search strategy (Additional file 1). The following keywords were employed in this search: “HIV,” “prevalence,” “men,” “men who have sex with men,” and “sub-Saharan Africa.” The published studies included did not deal with both the MSM and general male population simultaneously (Additionnel file 2). They provided the HIV infection prevalence among MSM. In an approach similar to that of Baral et al. [6], we consulted the Macro International database for Demographic and Health Surveys in each country in sub-Saharan Africa (http://dhsprogram.com). This led to estimates of the prevalence of HIV infection among men in the general population. For each study included, the DHS from its corresponding country was selected: this DHS was contemporary to the study on the prevalence of HIV among MSM (most often the same year or within one to 3 years of the selected MSM study). Otherwise, the grey literature was consulted, mainly UNAIDS and WHO periodic reports, but also monitoring reports of HIV interventions at country level, periodic progress reports on the fight against HIV in different countries, and reports on the national census of populations at a countrywide level to complete the information necessary to meet the objectives of this systematic review.

### Selection of articles

**Fig. 1:**
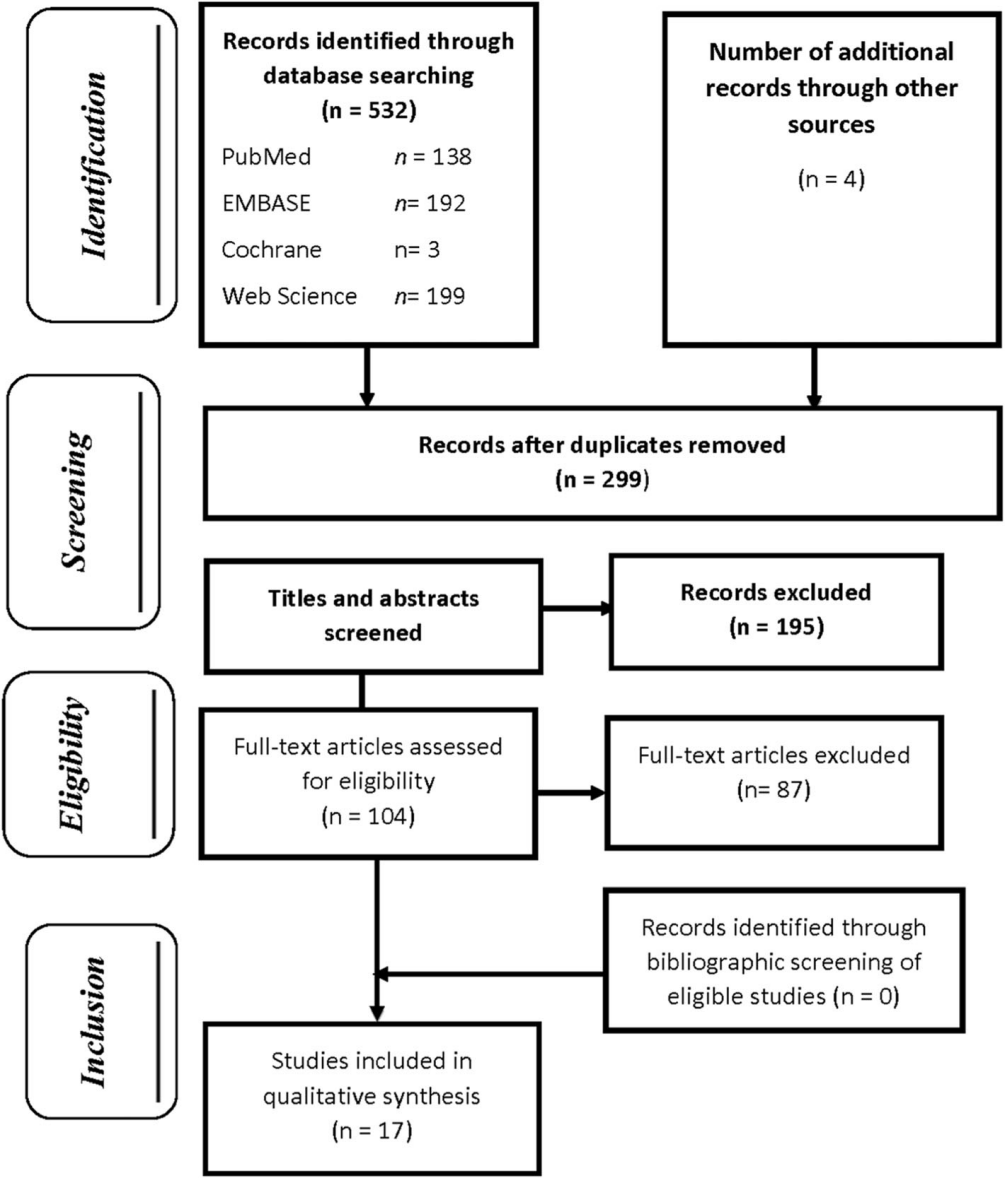
Flow chart of articles selection for the systematic review (PRISMA)

Two independent reviewers selected the articles based on the inclusion criteria; this was carried out in two stages. The first selection was made from the titles and abstracts of the articles. For a mutual and reproducible understanding between the two reviewers, a pilot test was carried out on approximately fifteen samples before the first selection (5% of the 299 articles randomly drawn after the elimination of duplicates). The inter-reviewer agreement represented by the Kappa coefficient was 74%. At the end of the first selection, 104 articles were selected out of the 299 initially pre-selected: the Kappa coefficient for the two reviewers was 92%. The second selection was carried out by reading the full text of the pre-selected 104 articles. The only articles selected were those that fully and completely met the inclusion criteria. This second selection was also preceded by a pilot test: it was carried out on 5 articles (5% of 104). The Kappa coefficient obtained was 85%. A summary of the key points was made to validate the final selection of articles by the two reviewers. The actual selection made it possible to definitively retain 17 articles; the inter-reviewer agreement was 71%. At each stage of the selection process, disagreements were resolved through discussion and consensus between the two reviewers (Fig. 1).

### Data collection

Data collection was carried out using a previously designed tool (Additional file 3). It was used to collect the following information: the identification of studies, target population, method used, software employed for data analysis, and results. This extraction was carried out independently by two (2) reviewers. Disagreements between the two were usually resolved through discussion based on the information to be retrieved as found in the data collection guide. The assessment of the risks of bias was performed using the Mixed Methods Appraisal Tool (MMAT) [8].

### Statistical analysis

For each MSM study included, we used HIV prevalence as reported for this population. HIV prevalence estimates in the general male population were taken from the results of contemporary DHS surveys in the same country (Table 1). To estimate the uncertainty associated with these measurements, estimates of the prevalence of HIV among MSM and men in the general population were used for each country with 95% confidence intervals (95% CI). The prevalence ratio (PR) was calculated by dividing HIV prevalence among MSM by that of men in the general population. Using meta-analysis, the pooled PR estimates of all the countries included were made using random-effect models. The heterogeneity between studies was assessed by examining the forest plot and Higgins I^2^ statistic. An I^2^ greater than 50% suggests high heterogeneity. Subgroup analysis was performed using PR estimates pooled according to the different geographic regions of sub-Saharan Africa (western, central, eastern and southern). Another subgroup analysis was performed according to the UNAIDS classification of the HIV epidemic level in the countries (mixed epidemic = prevalence < 1%; generalized epidemic = prevalence 1–5%; hyper-endemic = prevalence > 5%) [2, 42–44]. It is likely that MSM was included in some samples of men of childbearing age in the general population. Sensitivity analysis was performed to assess the impact of misclassification of exposure. This sensitivity analysis was carried out according to the approach used by Baral and al [6].. For each country, the total population of MSM (estimated from the sample) was subtracted from the general population of men of childbearing age. The prevalence of HIV infection among MSM was recalculated for a hypothetical population where MSM did not contribute to the prevalence of HIV in the general population. Descriptive analysis and meta-analysis were performed using the statistical software SAS version 9.1 and Revman 5.

**Table 1:** Characteristics of the selected studies LOCATION: After line 2 of page 8

Author	Country	Region	Number of MSM in the sample	Number of men in the general population	Sampling technique	HIV test from biological samples	Prevalence of HIV among MSM (%)	95% CI for MSM	Prevalence of HIV among men in the general population according to DHS (%)	95% CI for Men
Ekouevi D.K	TOGO	Lome, Aneho, Sokode-Kara	758	3987	simple random	Yes	19. 6[9]	15.9–23.8	1. 7[10]	1.2–2.2
Eduard J.	KENYA	Nairobi	285	2851	simple random	Yes	24.5 6[11]	7–34	4. 6[12]	3.5–5.6
Sidibe A.	SENEGAL	Dakar, Thies, Mbour, Saint Louis	463	3184	RDS	Yes	21. 5[13]	NR	0. 5[14]	0.2–0.7
Girault P.	GHANA	Greater Accra, Ashanti	166	3883	RDS	Yes	30. 3[15]	18.0–43.4	1. 1[16]	1.0–1.9
Mason K.	GAMBIA		207	3450	RDS	Yes	9. 8[17]	NR	1. 7[18]	0.9–2.4
Park J.	CAMEROON	Douala, Yaounde	511	6981	RDS	Yes	33.4 6[19]	NR	2. 9[20]	2.4–3.4
Hladik W.	UGANDA	Kampala	300	8673	RDS	Yes	13. 7[21]	7.9–20.1	6. 1[22]	5.5–6.7
Wirtz A.	MALAWI	Blantyre, Lilongue	338	6493	RDS	Yes	15.8 4[23]	7.2–17.6	8. 1[24]	7.1–9.2
Lahterta M.	MALI	Bamako	552	3503	RDS	Yes	13. 7[25]	NR	0. 8[26]	0.5–1.2
Dahoma M.	TANZANIA	Unguba Zanzibar	509	6865	RDS	Yes	12. 3[27]	8.7–16.3	4. 6[28]	3.7–5.4
Kendall C.	ANGOLA	Luanda	792	5144	RDS	Yes	3. 7[29]	NR	1. 9[30]	1.3–2.4
Baral. S.	SWAZILAND		324	3763	RDS	Yes	17. 6[31]	NR	19. 7[32]	17.9–21.5
Baral. S.	NAMIBIA	Windouk	218	3680	RDS	Yes	12.1 4[33]	8.7–17.14	10. 9[34]	9.2–12.6
Baral. S.	BOTSWANA	Gaborone	117	3763	RDS	Yes	19. 6[33]	13.5–27.8	19. 7[35]	17.9–21.5
Dahomah T.	BURKINA FASO	Ouagadougou	92	6314	convenience	Yes	14. 5[36]	NR	0. 8[37]	0.5–1.1
Stahlman S.	LESOTHO	MaseruMaputo	530	2646	RDS	Yes	32. 8[38]	NR	18. 6[39]	16.5–20.8
Hessou S.	BENIN	Cotonou, Calavi, Porto Novo, Parakou	364	3889	RDS	Yes	7. 7[40]	2.6–12.6	1[41]	0.69–1.31

95% CI: 95% confidence interval

## Results

The study selection process began on November 15, 2016. Five hundred thirty-six documents were found: 532 studies in electronic databases and 4 in UNAIDS reports. After eliminating the duplicates, 299 documents were retained. After reading their titles and abstracts and checking whether they met inclusion criteria, some studies were excluded (195). Thus, 104 articles were selected to be read in full. The reasons for exclusion were related to non-compliance with the target population, geographic area, exposure, type of estimate and frequency measurements calculated in the study. At the end of this process, 17 articles completely met the inclusion criteria and were selected (Fig. 1).

The characteristics of the studies included in this literature review are summarized in Table 1. The average prevalence rate of HIV was 17.81% among MSM (minimum, 3.7%; maximum 33.46%) and 6.15% among men in the general population (minimum, 0.5%; maximum, 19.7%).

All the studies were carried out in the capitals and/or major cities of the countries included. The Respondent-Driven Sampling (RDS) method was used by most of the studies (82.35%) to reach “hidden” populations such as MSM.

Regardless of the prevalence rate of HIV infection in the general population, type of epidemic and regional classification, the prevalence ratio of HIV infection among MSM compared with men in the general population varied from 0.89 (95% CI: 0.69–1.16) to 43 (95% CI: 25.56–72.34). Overall, the prevalence rate of HIV infection was 4.94 times higher among MSM than among men in the general population (95% CI: 2.91–8.37). Additionally, there was heterogeneity between studies: I^2^ = 98%, *p* <  0.00001 (Table 2).

**Table 2:** Prevalence ratio of HIV infection among MSM compared to men in the general population of countries in Sub-Saharan Africa

Country	N	Epidemic level	Prevalence Ratio*	95% CI
Senegal	3184	Generalized	43.00	[25.56–72.34]
Ghana	3883	Generalized	27.15	[18.68–40.62]
Burkina Faso	6314	Generalized	18.13	[8.97–36.14]
Mali	3503	Generalized	17.13	[11.21–26.17]
Cameroon	6981	Mixed	11.54	[9.61–13.85]
Togo	3987	Generalized	11.50	[8.46–15.62]
Benin	3889	Mixed	7.70	[4.80–12.36]
Gambia	3450	Generalized	5.76	[3.54–9.38]
Kenya	2851	Mixed	5.34	[4.10–6.95]
Angola	5144	Generalized	4.04	[2.67–6.10]
Tanzania	6865	Generalized	2.67	[2.08–3.44]
Uganda	8673	Generalized	2.25	[1.65–3.05]
Lesotho	2646	Hyper-endemic	1.76	[1.52–2.04]
Malawi	6493	Mixed	1.53	[1.14–2.06]
Namibia	3650	Hyper-endemic	1.14	[0.77–1.64]
Botswana	3763	Hyper-endemic	1.00	[0.69–1.45]
Swaziland	3763	Hyper-endemic	0.89	[0.69–1.16]
Total	79,069		4.94**	[2.91–8.37]

*n* = sample size of men by country

* Prevalence ratio of HIV infection among MSM compared with that among men in the general population

** *p* < 0.00001, *I*^2^ = 98%

95% CI: 95% confidence interval

Subgroup analyses indicated that in western and central Africa, the prevalence of HIV infection was 14.47 times higher in MSM than in men in the general population (95% CI: 9.90–21.13, *p* <  0,0000) [10, 14, 16, 18, 20, 26, 37, 45]. In eastern Africa, the prevalence of HIV infection was 3.39 times higher in MSM than in men in the general population (95% CI: 2.27–5.08; *p* <  0,00001). In southern Africa, the prevalence of HIV infection was 1.24 times higher among MSM than among men in the general population (95% CI: 0.91–1.69; *p* = 0.17) (Table 3).

**Table 3:** Prevalence ratio of HIV infection among MSM compared with that among men in the general population in countries in sub-Saharan Africa by region, prevalence level and type of epidemic

Parameters	Different levels	Number of countries	PR*[95% CI M-H random]	*p* value
	Western and Central	8	14.47 [9.90–21.13]	< 0.00001
	Eastern	4	3.39 [2.27–5.08]	< 0.00001
	Southern	5	1.24 [0.91–1.69]	0.17

**Fig. 2:**
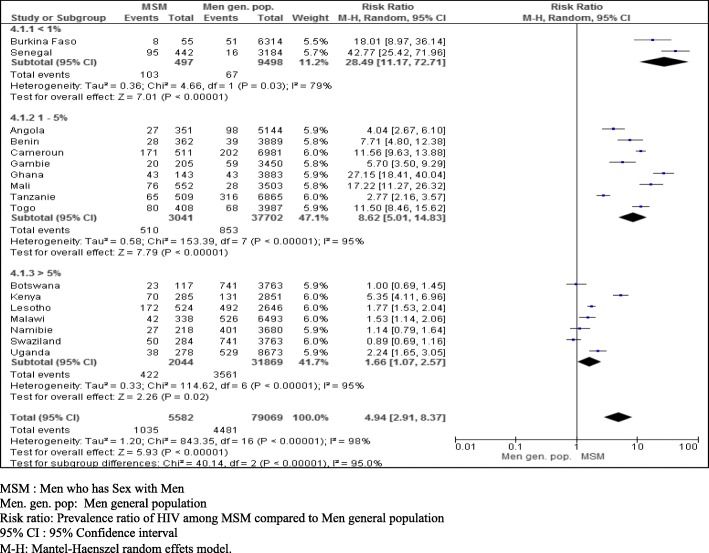
Prevalence ratio of HIV infection among MSM compared to men in the general population according to the prevalence levels of countries in sub Saharan Africa

The prevalence of HIV infection in low-prevalence countries (prevalence < 1%) was 28.49 times higher among MSM than among men in the general population (95% CI: 11.47–72.71, *p* = 0.03). In medium-prevalence countries (prevalence 1–5%), the prevalence of HIV infection was 8.62 times higher among MSM than among men in the general population (95% CI: 5.01–14.83, p <  0, 00001). In countries with a high prevalence (prevalence > 5%), the prevalence of HIV infection was 1.66 times higher among MSM than among men in the general population (95% CI: 1.07–2.52, *p* < 0, 00001) (Fig. 2).

**Fig. 3:**
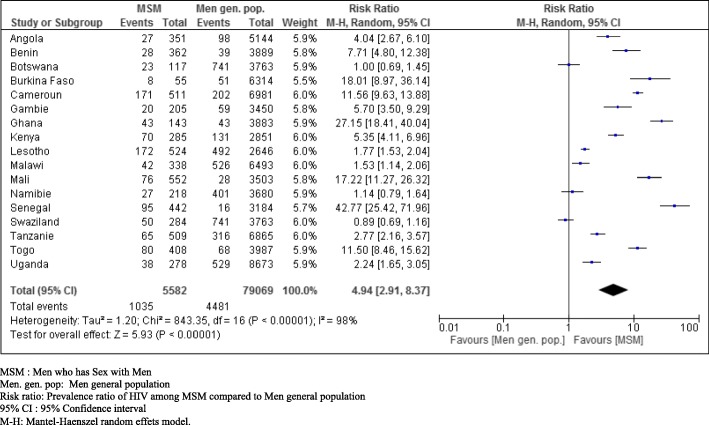
Assessment of the prevalence ratio of HIV infection among MSM compared to men in the general population and the prevalence of HIV infection among MSM compared to men in the general population of countries in sub Saharan Africa

Sensitivity analysis suggested that the prevalence of HIV infection was 4.54 times higher among MSM than among men in the general population, excluding MSM (95% CI: 2.66–7.75, p < 0, 00001) (Fig. 3).

## Discussion

To study the HIV epidemic among MSM and the link between this epidemic and its spread in the general population, we conducted a systematic review of studies on HIV among MSM in sub-Saharan African countries. Overall, most of the studies used RDS as a sampling technique to reach MSM. In total, the prevalence of HIV was five times higher among MSM than among men in the general population. The western and central regions of Africa, as well as low-prevalence countries (prevalence < 1%), had very high PR.

The data suggest that RDS is the most commonly used method for sampling MSM. To provide reliable estimates from a population that is difficult to access, this scientific method is also used to recruit MSM. This respondent-led sampling strategy is a probabilistic sampling method specifically designed to obtain samples from so-called “hidden” and socially organized populations. It is characterized by the sample to be studied being created by the MSM themselves through chain referrals [29, 46–48]. This is a variant of “snowball sampling;” however, unlike the latter, it has been shown that RDS produces unbiased estimates under certain conditions [49–53].

Regarding the regional analysis, the western and central regions of Africa had a very high PR because the national prevalence of HIV infection among men in the general population was quite low compared with that among MSM. According to the UNAIDS criteria, this situation makes MSM priority targets in these regions [2, 54]. The PRs are high in countries with mixed and generalized epidemics, while they are close to one in some of the massive hyper-endemic countries. Because the national prevalence in these countries was already very high, the prevalence of HIV among men in the general population was very close to that of MSM. In this context, the national response to HIV infection at the country level could also have a direct influence on the MSM subpopulation [2, 54]. We also noted that the PR was increased when the prevalence of HIV among men in the general population was decreased. This finding could be explained by the observation that, when the national prevalence is low, the prevalence in the MSM group is higher. Thus, MSM will constitute a high-risk group and will be a priority target of response measures. This systematic review draws its strength from the precision of the combined estimates of PR and a large aggregate size of samples of MSM and men in the general population. However, it is not without its limitations.

### Limitations

In general, access to MSM in many African countries has been difficult, particularly in terms of participation in studies because of discrimination and/or criminalization of their sexual orientation. The problems of participants‘safety and security in certain contexts would result in low levels of self-identification among MSM [6, 15, 55]. These barriers have likely limited the number and quality of studies and the availability of data on MSM in many countries in sub-Saharan Africa. Normally, the correct and efficient use of the RDS technique as a sampling method would help to partially solve this problem. Nevertheless, the literature also shows that RDS can give biased estimates when the recruitment does not completely follow the RDS principles in practice. This could be another limitation of our analysis [49–53].

Some of the studies included in this analysis used convenience sampling or a cross-sectional design and, therefore, may not be representative of MSM. To determine the risk of HIV infection among MSM in countries in sub-Saharan Africa, we used the DHS estimates of the prevalence in the general male population for each country as the unexposed population to calculate PR. However, this approach does not make the populations comparable and may have affected the validity of our results. MSM tend to congregate in urban areas, explaining, at least in part, why most of the reported studies were urban. This may limit the generalizability of the studies. Although 82.35% of the authors used the RDS method for sampling subjects, only half of them produced weighted data that considered the overall contribution of each referral chain [44, 56, 57]. This situation may affect the representativeness of the MSM in the studies. Otherwise, MSM are included in men of the general population. This could have increased estimates of HIV infection prevalence in the general population of men.

Selection bias is also possible because our research did not extend to unpublished articles and conference abstracts, which could affect the results of our systematic review. This could be partly responsible for the degree of associations observed in our study. To minimize this bias, DHS data and annual progress reports were sought to complement and refine the information provided by conventional databases.

The extent to which MSM are included, excluded or unidentified in these national estimates affects both their validity and the ability of our study to compare the prevalence of HIV among MSM with that among men in the general population. This may result in a classification bias or even an underestimation of the final result. However, the sensitivity analysis carried out for this purpose showed similar results in both groups.

## Conclusion

The results of the present study reveal the following: HIV prevalence is significantly higher in the populations of men who have sex with men than in men in the general population and, more specifically, in men in sub-Saharan Africa. This phenomenon was observed independently of the type of epidemic, geographical location and epidemic level. These MSM populations are known to be difficult to access because they live in autarky, hidden because of the extent of stigma and discrimination against them, especially in African countries, and this required a technique of sampling and recruitment adapted to reach the largest number of these MSM. This situation calls for new measures to address an effective and efficient response to HIV infection in this key population for universal access to prevention, treatment, care and support according to the WHO’s new 2015, 2016, 2017 guidelines for comprehensive and inclusive care of key populations. In addition, such populations in general, and particularly MSM, need to be integrated into epidemiological monitoring systems at the country level in sub-Saharan Africa. These innovations and actions can only provide substantive results if they are delivered in a socio-cultural and health environment of respect for gender identity and human rights. This way it will be possible to hope for a continuous and constant reversal of the trend of the HIV epidemic and achievement of the three 90x90x90 UNAIDS targets for sub-Saharan Africa.

## Supplementary information

**Additional file 1:** **Table S1.** Operationalization of the criteria for article selection by reviewers.

**Additional file 2:** **Table S2.** Detailed literature search strategies.

**Additional file 3.** Data collection guide.

## Data Availability

The dataset used and analyzed during the current study are available from the first author on reasonable request.

## Abbreviations

AIDS: Acquired Immune Deficiency Syndrome
DHS: Demographic and Health Surveys
HIV: Human Immunodeficiency Virus
MMAT: Mixed Methods Appraisal Tool
MSM: Men Who Have Sex With Men
PR: Prevalence Ratio
PRISMA: Preferred Reporting Items for Systematic Reviews and Meta-Analyses
RDS: Respondent-Driven Sampling Method
UNAIDS: The Joint United Nations Program on HIV and AIDS
WHO: World Health Organization
