# Supplementary material for: Comparison of the prevalence rates of HIV infection between men who have sex with men (MSM) and men in the general population in sub-Saharan Africa: a systematic review and meta-analysis
Source: BMC Public Health. 2019 Dec 4;19:1634. doi: 10.1186/s12889-019-8000-x (PMC6894288; doi:10.1186/s12889-019-8000-x)
Supplement: Supplementary file 1 — Additional file 1: Table S1. Operationalization of the criteria for article selection by reviewers. [file 12889_2019_8000_MOESM1_ESM.docx]

**Table S1:** Operationalization of the criteria for article selection by reviewers

| **Parameters** | **Informational content** | **Operationalization of the selection of titles and abstracts** |
| --- | --- | --- |
| P (Population) | Men | Does that article talk about men: biological males at least 18 years of age?  The first stage of the article selection will only involve abstracts, so if the age does not appear there, retain only N (the number of biological males). |
| I (Intervention / Exposure) | Having sexual intercourse with a man | All articles dealing with sexual relations/intercourse between men (biological males of same-sex) |
| C (Comparators / Comparison) | - Men (homosexuals) - Men & women (bisexuals) - Women (heterosexuals) | These men have a sexual orientation :   - These men have homosexual relations with each other - These men have sexual relations/intercourse with other men (homosexuals) - These men have sexual relations with other men AND with women (bisexuals) |
| O (Outcomes) | HIV (prevalence of HIV infection) | The article to retain must be related to this population, to this exposure and the outcome must be HIV infection or not (prevalence of HIV infection). |
| S (Study design / Articles to be included in the study) | Cross-sectional studies (descriptive and/or analytical studies); other reports and situational analyses conducted in Sub-Saharan Africa | All cross-sectional studies, in French or English, conducted in countries in Sub-Saharan Africa (descriptive and/or analytical studies) |

* Operationalization is a process of defining the measurement of a phenomenon that is not directly measurable, though its existence is indicated by other phenomena

**Table S2:** Detailed literature search strategies

| - **PubMed** | | | | | | | |
| --- | --- | --- | --- | --- | --- | --- | --- |
| - **N°** | - **Concepts** | | - **Search (Keywords)** | - **Date** | | - **Combination** | - **Results/articles** |
| - #1 | - Prevalence | | ("Prevalence"[TIAB] OR "Prevalence"[Mesh] ) | - 15 dec 2016 | |  | 545,768 |
| - #2 | - HIV | | ("HIV"[Mesh] AND "HIV Infections"[Mesh] OR "HIV"[TIAB] OR " AIDS"[TIAB] OR Human Immunodeficiency Virus [TIAB]) | - 15 dec 2016 | |  | 349,858 |
| - #3 | - Men who have sex with men, Men | | ("Homosexuality, Male"[Mesh] OR men who have sex with men [TIAB] OR MSM [TIAB] OR gay [TIAB]) | - 15 dec 2016 | |  | 19,892 |
| #4 | - Sub-Saharan Africa | | ("Africa South of the Sahara"[Mesh] OR "Africa South of the Sahara"[TIAB] OR Cameroon TIAB) OR Central African Republic [TIAB] OR Chad [TIAB] OR Congo [TIAB] OR Democratic Republic of the Congo [TIAB] OR Equatorial Guinea [TIAB] OR Gabon [TIAB] OR Burundi[TIAB] OR Djibouti[TIAB] OR Eritrea[TIAB] OR Ethiopia [TIAB] OR Kenya[TIAB] OR Rwanda[TIAB] OR Somalia [TIAB] OR South Sudan[TIAB] OR Sudan[TIAB] OR Tanzania[TIAB] OR Uganda[TIAB] OR Angola[TIAB] OR Botswana[TIAB] OR Lesotho[TIAB] OR Malawi[TIAB] OR Mozambique[TIAB] OR Namibia[TIAB] OR South Africa[TIAB] OR Swaziland [TIAB] OR Zambia [TIAB] OR Zimbabwe[TIAB] OR Benin[TIAB] OR Burkina Faso[TIAB] OR Cape Verde [TIAB] OR Cote d'Ivoire [TIAB] OR Gambia [TIAB] OR Ghana[TIAB] OR Guinea [TIAB] OR Guinea-Bissau[TIAB] OR Liberia[TIAB] OR Mali[TIAB] OR Mauritania[TIAB] OR Niger[TIAB] OR Nigeria [TIAB] OR Senegal[TIAB] OR Sierra Leone[TIAB] OR Togo[TIAB] OR Madagascar [TIAB]) | - 15 dec 2016 | |  | 322,787 |
| **#5** |  | |  | - **15 nov to 15 dec 2016** | | **#1 AND #2 AND #3 AND #4** | **138** |
| **EMBASE** | | | | | | | |
| - **N°** | | - **Concepts** | - **Search** | | - **Date** | - **Combination** | - **Results/articles** |
| - #1 | | - Prevalence | - 'prevalence'/exp OR 'prevalence':ti,ab | | - 15 dec 2016 |  | 772,099 |
| - #2 | | - HIV | 'human immunodeficiency virus'/exp OR aids:ti,ab OR HIV:ti,ab | | - 15 dec 2016 |  | 419,622 |
| - #3 | | - Hommes ayant des relations sexuelles avec des hommes, hommes | 'men who have sex with men'/exp OR 'men who have sex with men':ti,ab OR 'msm':ti,ab OR 'homesexuality':ti,ab OR 'men who have sex with men and women':ti,ab | | - 15 dec 2016 |  | 13,015 |
| #4 | | - Sub-Saharan Africa | - 'africa south of the sahara'/exp OR 'africa south of the sahara':ti,ab OR cameroon:ti,ab OR 'central african republic':ti,ab OR chad:ti,ab OR congo:ti,ab OR 'democratic republic of the congo':ti,ab OR 'equatorial guinea':ti,ab OR gabon:ti,ab OR burundi:ti,ab OR djibouti:ti,ab OR eritrea:ti,ab OR ethiopia:ti,ab OR kenya:ti,ab OR rwanda:ti,ab OR somalia:ti,ab OR 'south sudan':ti,ab OR sudan:ti,ab OR tanzania:ti,ab OR uganda:ti,ab OR angola:ti,ab OR botswana:ti,ab OR lesotho:ti,ab OR malawi:ti,ab OR mozambique:ti,ab OR namibia:ti,ab OR 'south africa':ti,ab OR swaziland:ti,ab OR zambia:ti,ab OR zimbabwe:ti,ab OR benin:ti,ab OR 'burkina faso':ti,ab OR 'cape verde':ti,ab OR 'cote ivoire':ti,ab OR gambia:ti,ab OR ghana:ti,ab OR guinea:ti,ab OR 'guinea-bissau':ti,ab OR liberia:ti,ab OR mali:ti,ab OR mauritania:ti,ab OR niger:ti,ab OR nigeria:ti,ab OR senegal:ti,ab OR 'sierra leone':ti,ab OR togo:ti,ab OR Madagascar :ti,ab | | - 15 dec 2016 |  | 350,191 |
| **#5** | |  |  | | - **15 dec 2016** | **#1AND #2 AND #3 AND #4** | **192** |
| **Cochrane** | | | | | | | |
| - **N°** | | - **Concepts** | - **Search** | | - **Date** | - **Combination** | - **Results/articles** |
| - #1 | | - Prevalence | - 'prevalence':kw,ti,ab | | - 15 dec 2016 |  | - 19,285 |
| - #2 | | - HIV | - HIV:kw,ti,ab OR aids:ti,ab OR 'Human immunodeficiency virus':ti,ab | | - 15 dec 2016 |  | - 16,909 |
| - #3 | | - Hommes ayant des relations sexuelles avec des hommes, hommes | - "homosexuality, male":kw OR MSM:ti,ab OR gay:ti,ab | | - 15 dec 2016 |  | - 597 |
| **#4** | |  |  | | - **15 dec 2016** | - **#1 and #2 and #3 and #4** | - **3** |
| **Web of Science** | | | | | | | |
| - **N°** | | - **Concepts** | - **Search** | | - **Date** | - **Combination** | - **Results/articles** |
| - #1 | | Prevalence | "Prevalence" | | - 15 dec 2016 |  | 601,615 |
| #2 | | HIV | "HIV" OR "AIDS" OR "Human Immunodeficiency Virus" | | - 15 dec 2016 |  | 407,719 |
| #3 | | Homosexuals, Men | "Homosexuality" OR "men who have sex with men" OR"MSM" OR"gay" | | - 15 dec 2016 |  | 37,140 |
| #4 | | - Sub-Saharan Africa | "Africa South of the Sahara" OR Cameroon OR "Central African Republic"OR Chad’’ OR Congo_OR "Democratic Republic of the Congo OR "Equatorial Guinea"__OR "Gabon"OR Burundi OR Djibouti OR Eritrea OR Ethiopia"OR Kenya OR Rwanda OR Somalia OR "South Sudan" OR Sudan OR Tanzania OR Uganda OR Angola OR Botswana OR Lesotho OR Malawi OR Mozambique OR Namibia OR South Africa OR Swaziland_OR Zambia_OR Zimbabwe OR Benin OR Burkina Faso OR Cape Verde__OR "Cote Ivoire"_OR Gambia_OR Ghana OR Guinea_OR "Guinea-Bissau" OR Liberia OR Mali OR Mauritania OR Niger OR Nigeria__OR Senegal OR "sierra Leone_" OR Togo OR Madagascar | | - 15 dec 2016 |  | 504,660 |
| **#5** | |  |  | |  | **#1 and #2 and #3 and #4** | **199** |
