# Supplementary material for: Comparison of the prevalence rates of HIV infection between men who have sex with men (MSM) and men in the general population in sub-Saharan Africa: a systematic review and meta-analysis
Source: BMC Public Health. 2019 Dec 4;19:1634. doi: 10.1186/s12889-019-8000-x (PMC6894288; doi:10.1186/s12889-019-8000-x)
Supplement: Supplementary file 3 — Additional file 3. Data collection guide. [file 12889_2019_8000_MOESM3_ESM.docx]

**Data Collection Guide**

**I‒ General Information**

| **Name** | **Types** | **Description** | **Modalities** | **Commentaries** |
| --- | --- | --- | --- | --- |
| 1. **General information** | | | | |
| Id | NUM | Identification number of the study |  |  |
| Auteur | ALPNUM | Name of the principal author | Text | Author’s surname and first names |
| Année | NUM | Year of publication | YY-MM-DD | Write only the year YYYY |
| Annrea | NUM | Year of completion of the study | YY-MM-DD | Write only the year YYYY.  If over two years, write the last year |
| Unipays | ALPNUM | The study took place in one country |  | Write the name of the country |
| Multipays | NUM | The study took place in only one country? | 1= Yes  2= No | Answer Yes or No.  If the study took place in one country, write the name of the country |
| Nomdespays | ALPNUM | If no, the study took place in two or more countries | text | Write the names of these countries (list of countries) |
| Région | ALPNUM | In which of the main region(s) of the country or countries did the study take place? | Text | Write the name(s) of this/these region(s) |

**II‒ Description of the target population**

| **Name** | **Types** | | | **Description** | **Modalities** | | **Commentaries** | |
| --- | --- | --- | --- | --- | --- | --- | --- | --- |
| 1. **Participants: Characteristics of men in the study population (homosexual, bisexual and heterosexual)** | | | | | | | | |
| **2.1 Homosexual, bisexual** | | | | | | | | |
| NbG_MSM | | NUM | Overall number of men who have sex with other men included in the study | | | ### | | Combined number of homosexuals and bisexuals |
| Nb_MSM | |  | Number of men who have sex with men included in the study (homosexuals) | | | ### | | Number of homosexuals |
| Profes | | ALPNUM |  | | | # | | Indicate the nature of the different professions of the targets |
|  |  |  | public officials | | | # | | 1= Yes 2= No |
|  |  |  | merchants | | | # | | 1= Yes 2= No |
|  |  |  | craftsmen / farmers | | | # | | 1= Yes 2= No |
|  |  |  | university students / students | | | # | | 1= Yes 2= No |
|  |  |  | without a profession / unemployed | | | # | | 1= Yes 2= No |
|  |  |  | others (specify) | | | # | | 1= Yes 2= No |
| Nivinst | | NUM |  | | | # | | Level of study reached by the participants in the three levels of education |
|  |  |  | primary | | | # | | 1= Yes 2= No |
|  |  |  | secondary | | | # | | 1= Yes 2= No |
|  |  |  | university | | | # | | 1= Yes 2= No |
|  |  |  | no formal education | | | # | | 1= Yes 2= No |
|  |  |  | other (specify) | | | # | | 1= Yes 2= No |
| MSM_ageM | | NUM | *Average age of MSM (Mean)* | | | ##.## | |  |
| MSM_ageSD | | NUM | *Standard Deviation of MSM age (SD)* | | | #.## | |  |
| MSM_ageMed | | NUM | *Median age of MSM* | | | ##.## | |  |
| MSM_ageMin | | NUM | *Minimum age of MSM* | | | ##.## | |  |
| MSM_ageMax | | NUM | *Maximum age of MSM* | | | ##.## | |  |
| Modsex | | NUM | Modality of orientation during sexual intercourse | | | # | | 1= Yes 2= No |
|  |  |  | insertive or active | | | # | | 1= Yes 2= No |
|  |  |  | receptive or passive | | | # | | 1= Yes 2= No |
|  |  |  | versatile or borderline | | | # | | 1= Yes 2= No |
| MSM_act | | NUM | Number of active MSM | | | ### | |  |
| MSM_pas | | NUM | Number of passive MSM | | | ### | |  |
| MSM_vers | | NUM | Number of versatile MSM | | | ### | |  |
| **2.2 The “heterosexual” men in the general population** | | | | | | | | |
| Nb_Hom | |  | Number of men in the general population included in the study (heterosexuals) | | | ### | |  |
| Profes_Hom | | ALPNUM |  | | | # | | Indicate the nature of the different professions of the targets |
|  | |  | public officials | | | # | | 1= Yes 2= No |
|  | |  | merchants | | | # | | 1= Yes 2= No |
|  | |  | craftsmen / farmers | | | # | | 1= Yes 2= No |
|  | |  | university students / students | | | # | | 1= Yes 2= No |
|  | |  | without a profession / unemployed | | | # | | 1= Yes 2= No |
|  | |  | others (specify) | | | # | | 1= Yes 2= No |
|  | |  | public officials | | | # | | 1= Yes 2= No |
| Nivinst_Hom | | NUM |  | | | # | | Level of study reached by the participants in the three levels of education |
|  | |  | primary | | | # | | 1= Yes 2= No |
|  | |  | secondary | | | # | | 1= Yes 2= No |
|  | |  | university | | | # | | 1= Yes 2= No |
|  | |  | no formal education | | | # | | 1= Yes 2= No |
|  | |  | other (specify) | | | # | | 1= Yes 2= No |
| Hom_ageM | | NUM | *Average age of men in the general population (Mean)* | | | ##.## | |  |
| Hom_ageSD | | NUM | *Standard Deviation of age of men in the general population (SD)* | | | #.## | |  |
| Hom_ageMed | | NUM | *Median age of men in the general population* | | | ##.## | |  |
| Hom_ageMin | | NUM | *Minimum age of men in the general population* | | | ##.## | |  |
| Hom_ageMax | | NUM | *Maximum age of men in the general population* | | | ##.## | |  |

**III‒ Methods**

| **Name** | **Types** | **Description** | **Modalities** | **Commentaries** |
| --- | --- | --- | --- | --- |
| 1. **Methods** | | | | |
| Objective | ALPNU | Present the main objective of the study | Text | Describe the main objective of the study |
| **Etud_Trans** | NUM | Check if the study design is cross-sectional: that it presents or describes the study data in an instantaneous way, like a photograph of the situation at the moment of the data collection | # | 1= Cross-sectional study  2= Other (specify) |
| TypeMSM_echant | ALPNU | Sampling method and type used to select MSM targets | # | 1= Convenience  2= RDS (Respondent-Driven Sampling)  3= Simple random sampling  4= Capture-recapture  5= Other (specify) |
| TypeHom_echant | NUM | Sampling method and type used to select men in the general population for the study | # | 1= Convenience  2= RDS (Respondent-Driven Sampling)  3= Simple random sampling  4= Capture-recapture  5= Other (specify) |
| Logi_analyse | NUM | The different types of software used for data analysis | # | 1= RDSAT  2=STATA  3=SAS  4=SPSS  5=RDSAT +STATA  6=RDSAT + SAS  7=RSDAT + SPSS  8= Other (specify) |
| Metest_VIH | NUM | Method used to collect the data from HIV test | # | 1= Biological test  2= Self-reporting  3= Unclear |
| TestVIH_val | NUM | The procedure for carrying out the HIV test is validated according to WHO/UNAIDS standards and/or national screening policy at the country level | # | 1= Yes  2= No  3= Unclear  4= Other (specify) |

**IV‒ Intervention / Exposure**

| **Name** | **Types** | **Description** | **Modalities** | **Commentaries** |
| --- | --- | --- | --- | --- |
| 1. **Exposition** | | | | |
| Def-expo | ALPNUM | Definition of exposure: men who had sexual intercourse with men at least once in the 12 months prior to the study | # | 1= Yes  2= No  3= Other (specify)  4= Unclear |
| Expo1 | NUM | He only has sexual relations with a man or other men (homosexual) | # | 1= Yes  2= No  3= Unclear |
| Expo2 | NUM | He has sexual intercourse with a man or men as well as with a woman or women (bisexual) | # | 1= Yes  2= No  3= Unclear |
| Expo3 | NUM | He only has sexual relations with a woman or women (heterosexual) | # | 1= Yes  2= No  3= Unclear |

**V‒ Results**

| **Name** | **Types** | **Description** | **Modalities** | **Commentaries** |
| --- | --- | --- | --- | --- |
| 1. **Results** | | | | |
| **5.1 Frequency measurement** | | | | |
| PrevGHIV_MSM | NUM | Proportion of HIV infection in the entire MSM group (homosexuals and bisexuals) | ##.## |  |
| PrevHIV_MSM | NUM | Proportion of HIV infection in the MSM group (homosexuals) | ##.## |  |
| PrecHIV_MSMb | NUM | Proportion of HIV infection in the MSM group (bisexuals) | ##.## |  |
| PrecHIV_Hom | NUM | Proportion of HIV infection in the group of heterosexual men in the general population | ##.## | This information often exists in other studies completely distinct from that of MSM in terms of sample size, namely DHS. The most difficult part is that some countries are still at the level of carrying out sentinel surveillance among pregnant women to estimate their national prevalence (see Prof. Alary to decide) |
| **5.2 Measure of association** | | | | |
| Rap_Prev | NUM | Prevalence Ratio of HIV infection among MSM compared to men in the general population | ##.## | Getting the denominator of the fraction is difficult because this information is often in a different study |
| Inter_conf | NUM | Calculate the different Confidence Intervals | ##.## |  |

**VI‒** C**riteria for quality assessment**

| **Name** | **Types** | **Description** | **Modalities** | **Commentaries** |
| --- | --- | --- | --- | --- |
| 1. **Cross-sectional study, descriptive or not** | | | | |
| **6.1 Is the sampling strategy appropriate to answer the quantitative research question?** | | | | |
| Strat_ech | NUM | See if the authors used the most appropriate and accepted sampling strategy, the one found in the literature review for the selection of MSM. (RDS) | # | 1= Yes ( RDS)  2= No  3= Unclear |
| **6.2 Is the sample representative of the population?** | | | | |
| Crit_Inclus | NUM | The inclusion and exclusion criteria exist and are well defined and explained | # | 1= Yes (inclusion and/or exclusion)  2= No  3= Unclear |
| Non_part | NUM | The reasons and motives why some of the participants chose not to participate in the study are explained and presented | # | 1= Yes (well explained and presented)  2= absent (not presented or explained)  3= Unclear |
| **6.3 Are the measurements appropriate (clear origin, known validity or standard instrument)?** | | | | |
| Mes_just | NUM | The measurements are justified and appropriate to answer the research question | # | 1= Yes  2= No  3= Unclear |
| Bon_mes | NUM | Do the measures reflect what they are supposed to measure? | # | 1= Yes  2= No  3= Unclear |
| **6.4 Is there an acceptable response rate (60% or more)?** | | | | |
| Test_VIH | NUM | Is the rate of voluntary participation to HIV testing superior or equal to 60% of the starting sample included in the study? | # | 1= Yes  2= No  3= Unclear |
